# Healthier Lives Implementation Research Network for Māori and Pacific community health providers in Aotearoa New Zealand: a study protocol with an observational mixed methods design

**DOI:** 10.1186/s43058-022-00373-4

**Published:** 2022-11-22

**Authors:** John Oetzel, Dianne Sika-Paotonu, Darrio Penetito-Hemara, Akarere Henry

**Affiliations:** 1 University of Waikato, Hamilton, New Zealand; 2 University of Otago, Wellington, New Zealand; 3 Te Hotu Manawa Māori Trust trading as Toi Tangata, Auckland, New Zealand; 4 South Waikato Pacific Islands Community Services, Tokoroa, New Zealand

**Keywords:** Learning collaborative, Implementation barriers and facilitators, Health equity, Māori health, Pacific health

## Abstract

**Background:**

Despite incremental gains in the Aotearoa New Zealand health sector, Māori and Pacific peoples still experience poorer health outcomes than non-Māori and non-Pacific. Access to the latest research and innovation is critical to improving and addressing health outcomes and health inequities in particular. However, there are numerous challenges to translating research into practice including that there is currently no known a specific infrastructure in Aotearoa New Zealand to facilitate this process. The aim of the project is to develop a network of community providers, researchers and health systems representatives that can help facilitate the implementation of novel and innovative programmes and products that help to meet the health needs of Māori and Pacific communities.

**Methods:**

This project has three stages, one of which has been completed. In Stage 1, we engaged with key leaders of organisations from various components in the health system through a co-design process to identify parameters and infrastructure of the network. In Stage 2, we propose to construct the network involving approximately 20–30 community providers (and other affiliated researchers and health system representatives) and refine its parameters through an additional co-design process. Additionally, we will use a mixed methods research design using survey and interviews to identify perceived implementation needs, facilitators and barriers to help inform the work in the third stage. In Stage 3, we will support the active implementation of evidence-based programmes with a smaller number of providers (approximately four to eight community providers depending on the complexity of the implementation). Mixed methods research will be conducted to understand facilitators and barriers to implementation processes and outcomes.

**Discussion:**

The proposed network infrastructure is an equity-oriented strategy focused on building capacity through a strength-based approach that can help address inequities over time. Our “proof-of-concept” study will not be able to change inequities in that time period given its relatively small scale and time period, but it should set the foundation for continued equity-oriented work.

**Supplementary Information:**

The online version contains supplementary material available at 10.1186/s43058-022-00373-4.

Contributions to the literature

1. This study describes a network that focuses on the translation of research into practice and seeks to help address health inequities for Māori and Pacific communities in Aotearoa New Zealand; there is no known infrastructure currently to address these issues.
2. The study illustrates how a co-design or collaborative implementation process works to address health inequities for Indigenous communities by including community health providers as implementation partners rather than just sites of implementation.
3. The study includes research to identify implementation barriers, facilitators and outcomes, which are understudied in Indigenous contexts.

## Background

Health inequities experienced by Māori (Indigenous peoples) and Pacific peoples in Aotearoa New Zealand within the context of colonisation are well documented [1, 2]. Despite incremental gains in the New Zealand health sector, Māori and Pacific peoples still experience poorer health outcomes when compared with non-Māori/non-Pacific population groups, ultimately living shorter lives and dying earlier from preventable diseases [1, 2]. Life expectancy for Māori is 7 years less than for other Aotearoa New Zealanders with major causes of death (e.g. cardiovascular disease, cancer, type 2 diabetes) being preventable and treatable [3]. Life expectancy for Pacific people is approximately five years less than other Aotearoa New Zealanders also with higher rates of premature death caused by cardiovascular disease, diabetes and cancer [3]. Some of the key determinants for these health outcomes include differential exposure to determinants of health such as level of deprivation and socioeconomic differences, inequitable access to health and social systems, colonial history, not following the principles of Te Tiriti o Waitangi (Treaty of Waitangi; Treaty that founded Aotearoa New Zealand and guaranteed Māori rights) and racism [3–5].

It is widely understood that having access to the latest research and innovation is critical to improving adverse health outcomes and addressing health inequities in particular [6, 7]. However, there are numerous challenges that exist including the fact it takes many years before scientific discoveries are translated into real-world practices, and only a very small number (roughly 15%) ever reach that point [8–10]. A further challenge that contributes to health inequities in Aotearoa New Zealand is that best clinical practices and health programmes (used instead of interventions) are not equally translated to Māori and Pacific communities [1, 3] and/or are translated in a way that is timely, cultural appropriate and acknowledges self-determination [11].

Scholars suggest that to address these inequities there is a need to develop infrastructure that provides translational pathways for making research accessible, useable and relevant for those organisations to help implement new knowledge and practices within communities [12–14]. An example of one such structure is a network or learning collaborative that develops partnerships amongst communities, community providers/practices, researchers, and representatives from various points in the health system. For example, practice-based research networks (PBRNs) are networks of primary care practices that collaborate, often with research institutes, to undertake research activities and quality improvement activities aimed at better understanding and improving primary care services and patient outcomes [15, 16]. Another structure example is the US National Institute for Health’s National Center for Advancing Translational Research’s Clinical and Translational Science Awards (CTSA). The CTSA program was started in 2006 to expedite the translation of science from basic discoveries to real-world solutions. In 2022, the CTSA Consortium recommended several goals necessary to achieve diversity, equity, and inclusion in clinical and translational research, including funding health equity and community-oriented research at parity with biomedical research [17].

A third type of infrastructure, and the focus in the current study, is the learning collaborative (LC). A LC is a network of participants (individuals, organisation, systems) that share information, build capacity to address a common health issue(s), and conduct work or implement programmes on the health issue(s) [18–20]. LCs have been frequently used with some positive outcomes demonstrated in recent research including developed research capacity and patient/community outcomes [18, 19]. LCs are flexible structures which benefit implementation in a particular context, but also can be a challenge in identifying the core structures. To address this issue, Nix and colleagues developed a taxonomy of four primary elements: innovation, social systems, communication and time [20]. Innovation refers to the novel components that are being implemented. Social system refers to the collaborative environment and structure including both elements of the network infrastructure and the larger health system. Communication covers how information is shared amongst network members and also how the common vision and goals are identified. Time relates to the development and duration of the LC. Further research suggests additional structures: (a) a focus on systems; (b) engagement of local and interdisciplinary teams to assume ownership of the implementation project; (c) centralised infrastructure to support the implementation work; (d) encouraging local adaptation of the programme; and (e) create of a participatory/collaborative culture within the LC and larger system [15, 16, 21, 22].

While the infrastructure is an important element for facilitating the implementation of novel programmes, it is necessary to understand the needs, facilitators and barriers of implementation projects. The Consolidated Framework for Implementation Research (CFIR) integrates 19 different models of implementation science and thus provides a comprehensive and inclusive framework that has been frequently utilised in implementation contexts [23, 24]. CFIR includes five elements: intervention, the individuals involved, inner setting, outer setting, and process [23]. *Intervention* incorporates aspects of the intervention itself such as novelty, compatibility, relative advantage, and supporting evidence; and whether the intervention has been adapted to local contexts [24]. *Individuals* are the intervention implementers, including their skills and characteristics including cultural values, experience, and affiliations. The *inner setting* refers to the organisation implementing the intervention and the level of managerial support. The *outer setting* encompasses larger economic, health, social, and political contexts in which the organisation operates. *Process* comprises the implementation methods and means [23].

A couple of studies provide a foundation for understanding implementation facilitators and barriers in Aotearoa New Zealand. The first study examined the facilitators and barriers of implementing a novel health programme developed by another organisation (creator) [25]. The programme was evidence-based and developed in partnership with a Māori community provider. The study included 17 interviews with health and social service providers with experience working with Māori communities and implementing health programmes. The findings illustrated three key facilitators: a) community engagement (the need to build relationships with community members and not just the providers); b) programme structure and creator’s experience (have a guiding framework and the experiences of the creators help facilitate use of the programme); and c) programme adaptability (being able to adapt the programme to fit the local cultural context). The barriers related to the need for funding, funding constraints (limiting innovation), and organisational constraints (research capacity and staffing).

The second study described the co-design and implementation processes utilised in translating an evidenced-based peer-education programme for older Māori to new communities [26]. The original research involved a peer-education programme for kaumātua (elders) working through later-stage life transitions that were co-developed by a Māori community provider and a group of university researchers [27, 28]. The study used a co-design process with five additional Māori community providers to adapt and implement the programme with their respective kaumātua [29]. Data were collected from research documentation, community meeting and briefing notes, and interviews with community researchers. Key lessons learnt include the following: (a) *Have structure with flexibility*—Māori community providers liked having a structured programme to start, but one that allows flexibility in implementation process and programme features to fit their local context; (b) *Use a participatory community engagement or co-design approach—*community providers know their communities and can thus enhance the adaptation of the programme. They also need to engage with their community members directly to ensure interest and benefit; (c) *Provide financial and/or human resources to support implementation—*to adequately participate in the co-design and implementation process, community providers need financial and human support resources during the implementation process; and (d) *Integrate key knowledge and end users through advisory boards*—advisory board members provide strong feedback about the project, but also are well placed to support additional implementation, dissemination and advocacy.

### Aims

The expected outcomes of this study include a network that centres and connects Māori and Pacific community providers and includes researchers and key partners from within various components of the health system. The network should add value for all participants by providing an infrastructure that facilitates research translation into practice to help address health inequities within these communities through a participatory or co-design approach. The research outcomes are to provide an assessment of the value of the network and also share key lessons learnt from implementation processes and outcomes. With this grounding, there are two specific aims for this project work:

- To develop a provider-based implementation research network to facilitate research translation into practice to help address inequities in health for Māori and Pacific communities.
- To identify implementation needs, experiences, processes and outcomes of network members, particularly Māori and Pacific community providers who are either contemplating or actively trialling the implementation of novel health programmes.

## Research design

This study relates to Stage Two and Three in the development of a learning collaborative network called the Healthier Lives Implementation Research Network (with opportunity for re-naming during a later co-design process). Stage One included some preliminary research and involved a co-design process in defining the parameters and structures of the network with kaupapa partners (i.e., stakeholders). Stage Two refers to the construction of the network and research to assess implementation needs. Stage Three refers to active implementation of programmes with network members and research about processes and outcomes of this implementation. The overall research design during this stage is concurrent mixed methods using interviews, focus groups, and surveys (and potentially other data as determined during the co-design process). Both Stage Two and Three along with specific research methods will be co-designed with network members following the He Pikinga Waiora (Enhancing Wellbeing; HPW) framework and research on learning collaboratives [30, 31]. HPW is grounded in Indigenous knowledge, participatory approaches and systems thinking.

### Stage One: feasibility and co-design with kaupapa partners

Given the needs to address health inequities in Aotearoa New Zealand from a strengths-based perspective, the learning collaborative network appeared to be a potential infrastructure to help translate research into practice in order to enhance health outcomes and advance health equity. We completed some research a study to explore the feasibility of constructing this network [32]. We conducted 24 interviews with participants from various regions in Aotearoa New Zealand (Auckland, Waikato, Bay of Plenty/Lakes, Wellington and Otago). Participants included CEOs, General Managers, General Practitioners, Nurses, Practice Mangers from practices or community providers with a patient population at least 50% Māori or Pacific attendees. There were also three researchers who worked with Māori or Pacific patients or community members.

We found overwhelming support for the establishment of the network. Participants indicated that the network offered an opportunity for shared learning and collaboration, translation of research into practice, and a safe space to innovate. They also acknowledged constraints that included inadequate resourcing and staffing, limited time, and negative experience with prior research efforts. They suggested that the network would help address some of these challenges through strong community engagement and through a participatory approach, a commitment to capacity building and adding value to the providers, linking/aligning with other organisations to support impact and sustainability, and a focus on addressing holistic community outcomes for Māori and Pacific communities.

With the feasibility of the network established, we engaged with kaupapa partners about identifying the parameters of the network. The primary engagement was a co-design hui/fono (meeting). We also had initial scoping conversations with some who also attended the co-design hui/fono and a few who could not attend during the specified times. Furthermore, we had debriefing conversations to reflect on learnings with some partners. The co-design hui/fono was a virtual 2-day event over 4 h (2 h each day). Overall, we asked participants to consider three key questions: (a) What are key challenges and how can the network be successful? (b) What should the network look like? (c) What are the structures for the implementation research network?

The kaupapa partners were leaders in a variety of organisations that might directly participate in the network or key links into the health system (e.g., CEOs/General Managers Māori and Pacific community providers, directors of Māori and Pacific Health from District Health Boards and Primary Health Organisations, Ministry of Health, Māori Health Authority, leaders from the health-oriented National Science Challenges). This broad scope helped to identify key challenges and opportunities of factors in the system that may be facilitators or barriers to an effective network. We had a total of 25 unique people participate in the co-design hui/fono, 16 participate in initial scoping conversations, and five participate in debriefing conversations.

Overall, we concluded that there was support for the network. Several key themes achieved consensus during the conversations: (a) the need to have a clear focus for the network and to co-design this focus with the community providers; (b) having a strong infrastructure to support the community providers involved including a strong communication component for sharing the vision and building relationships; (c) identify expectations of participants and ensure there is value added for participants; (d) having touchpoints/linkages with key organisations in the health system and to maintain flexibility given the vast restructure to the health system in Aotearoa New Zealand; (e) having an action-oriented focus on implementing programmes although with some screening of the evidence supporting specific programmes; and (f) a need to think about sustaining the network beyond the initial funding period. Two points did not achieve consensus during the hui/fono: (a) whether it should be a single network or separate Māori and Pacific provider networks; and (b) the size and scope of the network (whether national or regional focus). We identified a focus, framework/principles, and infrastructure that reflects the points of consensus and also provides a middle ground that we think integrates the different perspectives on two areas not achieving consensus. These elements have been shared with the original invitees for review and validation and Supplemental file 1 includes the final version. Only minor changes were suggested in the validation process.

The central structure includes four co-directors. One director (JO) will oversee the relationships with researchers and health system participants, establish an advisory board, lead a communication team and seek additional funding for sustainability. The other three directors will be responsible for liaising with Māori (DPH) and Pacific community providers (DSP, AH); we will have separate branches to support the unique cultural communities and yet have a central infrastructure to avoid duplication. These branches are supported with a community researcher each.

### Stage Two: constructing the network

The second stage is developing the network based on these parameters and structures. It will take place over roughly nine months to ensure the focus is clear and the network will meet the needs of the members, particularly the community providers. It also needs to be responsive to the changes in the current health system. The primary work will be constructing the network, co-designing the network, research to assess implementation needs and sharing implementation opportunities.

#### Constructing the network

Invitations to participate in the network will be led by the co-directors with the support of the community researchers. Initial contact will be made to gauge interest in participating as part of the network. Contact will be made through the leadership team networks, the networks of Healthier Lives National Science Challenge researchers and other National Science Challenges with a health focus). Participants will include community providers, researchers, and key touchpoints within the health system. We had nearly 40 participants who were interested in participating in the co-design hui/fono and being involved in the network; these include 10 community providers along with another 20 providers from our preliminary research work showing interest in participating in the network signalling the foundation for a sufficient network size.

We will also invite six to eight members to the advisory board. The participants from the co-design hui/fono are logical choices to be a part of this group given their expertise and interest. We will select these members during this construction stage to meet the needs of the network as they evolve as well as be responsive to the changes in the health system in Aotearoa New Zealand. As a result of a comprehensive review of the health system, major changes to the structure and focus of the system have been undertaken [33]. In brief, in recent years, the Ministry of Health stewards the health system which included 20 District Health Boards (DHBs). Māori and Pacific community providers offer a variety of health services guided by Māori and Pacific worldviews focusing on holistic well-being and receive their contracts from the DHBs. In the new system, the Ministry of Health will continue to steward the health system although with a much stronger focus on health equity, prevention and determinants of health. There will be two arms that commission and manage health services: Health New Zealand and the Māori Health Authority (MHA). Māori community providers will likely receive their contracts from the MHA and possibly Health New Zealand; Pacific community providers from Health New Zealand. These changes were implemented on 1 July 2022 although are still evolving. There will also be a locality approach emphasising public health prevention for every geographic area with roll out over the coming years.

#### Co-designing the network

During the invitation process, we will explain to the providers of the interest in co-designing the network with participants. We want to build trusting relationships with the members and help them meet the needs of their communities. This participatory process will begin by reviewing the outcomes and proposed structure of the first stage and revising and developing the focus, framework/principles and infrastructure. This process will be guided by our initial framework and principles and include conversations with network members and an additional co-design hui/fono to formally review these elements and identify research needs. We will apply procedures similar to those outlined in the first stage given the successful outcome of that process. We will refine the network structure, communication methods, principles and focus as a result.

#### Assessing implementation needs

We will use a concurrent mixed-methods research design [34] to identify implementation needs, facilitators and barriers of network members. Overall, the research focus and processes will be co-constructed with network members and particularly the community providers. We also hope to identify research gaps and then put those gaps out to the researchers in the network (i.e., providers identify a need and no programmes exist). The proposed research methods are a starting point for conversations and can be revised during the co-design process.

##### Participants

Participants will be network members including representatives from community providers, researchers, and people within the health system. We will have two types of participants: (a) those who are loosely involved in the network or considering participating; and (b) those who are closely connected network members. We aim to have approximately 50 participants who are loosely involved and 30 who are closely involved complete a survey to explore general perceived patterns in needs, facilitators and barriers of implementation. The proposal sample size was determined by a balance of resources and power to determine the optimal design and confirmed with NCSS-PASS software [35]. This sample size is sufficient to detect a medium effect size (*r*=.3, power=.80, *p*=.05) in associations with perceived outcomes. Participants who are closely involved will also participate in an in-depth interview to explore specific needs related to their communities (*n*=15).

##### Measures

For the survey, we will include measures that reflect the CFIR and HPW frameworks on key components about implementation process. We will use measures that we have employed in an Aotearoa New Zealand sample [36] based on an international review of implementation measures [37]. These include measures of the innovation, the inner setting/organisation, outer setting/system, individuals, and process as facilitators and barriers to implementation effectiveness. Surveys will be limited to approximately 30–40 items to reduce burden for the participants.

For the interviews, we have developed interview/focus group protocols around key HPW processes that will be utilised for this study [38]. We will also include questions that assess implementation needs for providers around capacity, funding, and health issues. Finally, we will also assess readiness to implement around health equity [38, 39] adapted to this context. For all measures and protocols, network members will identify whether the tools should be translated into te reo Māori or specific Pacific languages as well. If so, we will use and translation and back translation process.

##### Procedures

Participants who are closely involved will participate in semi-structured, in-depth interviews conducted by the community researchers. The interview will take place either via zoom or ideally face-to-face in a location chosen by the participant. A focus group may be used if there are multiple participants in a single organisation. All interviews/focus groups will be recorded and transcribed with the permission of the participants. The survey will be created and administered via Qualtrics. Participants can request that the community researcher read the questions to them or complete it on their own time. Participants will receive a $50 koha, meaalofa, mea'ofa (gift) for participating in either the survey or interview/focus group.

##### Data analysis

We will analyse the qualitative data using thematic analysis [40]. Each analysis will be undertaken by two research team members, with at least one Māori and one Pacific team member for those respective analyses [41, 42]. We will also explore including network members in this phase of analysis. At the very least, themes will be shared with network members as a validity check with revisions to the analysis and interpretation resulting from this sharing.

Quantitative data analysis will include several steps. First, prior to conducting the primary data analysis, we will establish the psychometric properties of specific scales within the project. Factorial validity will be assessed with confirmatory factor analysis (using a copy method to gain sufficient sample size) and reliability established with Cronbach’s alpha. Second, statistical assumptions including patterns of missing data will be assessed. Third, descriptive statistics including means, standard deviations, and confidence intervals for continuous data and frequencies for categorical data will be provided. Finally, the data will be analysed through correlational analysis and multivariate regression models, using mixed models if we have multiple respondents within organisations. Analysis will be undertaken with SPSS and AMOS 28 [43, 44].

##### Cultural safety and research ethics

The research team has been granted ethics approval through the University of Waikato’s Human Research Ethics Committee (Health2022#45). We are mindful that the projects involve sensitive topics, and we will utilise our prior experience and that of the wider team to protect participants’ confidentiality and honour their status. We will use culturally appropriate approaches for data collection developed in earlier projects that our team members have used previously (adapting based on network member feedback) [42, 45–47].

#### Sharing implementation opportunities

The final component in the Stage Two is to identify potential programmes or products for implementation and share these opportunities with providers. We will solicit these programmes and provide a two-page research brief that identify the evidence and brief description, and resources available and needed for implementation. We will determine if these briefs are the best way to communicate the opportunities with community providers or whether there are other preferences (e.g., short videos, longer reports, workshops/webinars). We will also seek out additional opportunities from researchers associated with the national science challenges and other researchers with other funding sources. The opportunities will be reviewed by the advisory board to determine if they should be shared with the network.

As part of the sharing process and preparing for the third stage, we envision holding workshops and webinars that share implementation programmes and also help network members prepare for implementation. These workshops might focus on understanding particular programmes, help develop research capacity for network members interested in implementation research (for community providers and researchers with limited experience in implementation), and grant and research opportunities. As part of these workshops and through the research assessing implementation needs, we may identify needs without known programmes; these workshops might enable a provider to connect with researchers and develop a programme that is community driven rather than researcher driven.

### Stage Three: active implementation

The final stage involves community providers actively adapting and trialling programmes. We are open to adaptation of the programmes within functional equivalence to enhance acceptance and ownership. Thus, like the other stages, the active implementation will be completed through a participatory approach. There are three components to this stage: implementation support, implementation research and sustainability.

#### Implementation support

The first step in this process will be identifying community providers that are ready, have identified a programme that matches their need, and have the capacity (with support) to implement the programme. Funding will be provided to implement the programme as a pilot trial (i.e. small-scale implementation relative to need and capacity). The number of pilot trials will be dependent on funding available, costs of implementation, number of interested providers and our capacity to support the implementation.

After identifying the providers and programme, the community researchers and leadership team will help the providers work through the process of adapting the programme if needed. After the adaptation process, the community researchers will support the implementation process with the providers (training, supporting administration, etc.). We will also invite the original creators to participate in the co-design and implementation process as network members—this is one of our expectations of researchers participating in the network.

#### Implementation research

We will research the implementation process and outcomes of the projects using a concurrent triangulation mixed-methods approach [34]. The primary design within this overall approach will be pre-test/post-test only for a single group. We are not employing more rigorous hybrid effectiveness designs due to financial and time constraints as well as the fact that these programmes already have an evidence base to support their use. The goal of this research is to understand facilitators and barriers to effective implementation processes and outcomes. Similar to the previous stage, the research focus and processes will be co-constructed with network members and particularly the community providers. Again, we offer preliminary research methods as a starting point for conversations and to provide reviewers with the foundation of the research.

##### Participants

There will be two types of participants for the implementation trial: those participating in the implementation process and community members receiving the programme. We anticipate to be working with four to eight total providers in the implementation trials each with several participants engaged in the adaption and implementation of the programme (depending on the complexity of each implementation). We anticipate small-scale trials of roughly 20–30 participants each. Given that these programmes have evidence of effectiveness, we anticipate medium to large effects; thus, this sample size is sufficient to detect these effects with a pre-test/post-test design [35].

##### Measures

For those participating in the adaptation and implementation of the programme, we will use the interview/focus group protocols around key HPW processes to explore the implementation process and satisfaction with the process [38]. We will also include questions about details as to the exact adaptations made; these adaptations will also be identified through meeting minutes and changes to guidelines [26, 48, 49].

For the patients, we will include outcome measures related to the specific programmes. As these will not be selected until Stage Three, we cannot provide specific measures; however, they may include weight, body-mass index, HbA1c, health-related quality of life and other outcomes. We will also include survey measures of participation in the programme (attendance, use of the product, satisfaction). For all measures and protocols, we will identify from network members whether the tools should be translated into te reo Māori or specific Pacific languages. If so, we will use a translation and back translation process.

##### Procedures

Participants who are involved in the implementation process will participate in semi-structured, in-depth interviews similar to the procedures identified in the second stage. Participants in the programme will complete surveys similar to the procedures in the second stage. Any clinical outcomes will be collected during regular visits with a nurse or doctor. Individual surveys will be conducted by the community researchers or leadership team. Participants will receive a $50 koha (gift) for participating in the data collection. Cultural safety and research ethics procedures identified in Stage Two will also apply to this stage.

##### Data analysis

We will analyse the qualitative data using thematic analysis following procedures in the second stage. Quantitative data analysis will follow similar procedures as well except that the final analysis will be based on paired sample t-tests to identify differences before and after participation.

#### Sustainability

The proposed funding will support the network until June 2024. However, if the network is well received and effective, we will aim to sustain the network for a longer-time period. To support sustainability and consistent with the HPW framework (i.e., integrated knowledge translation), we have included kaupapa partners and end users early in the development of the network (i.e. Stage One). These partners can see the value of the network and have offered to support it. For example, the Ministry of Health Long-term Conditions provided financial support for this project in addition to the primary funding by the Healthier Lives National Science Challenge. We also have identified the need to seek additional funding and one of our directors (JO) will be the lead in writing grants to support the long-term sustainability of the network.

During this final stage and in support of the effectiveness of the network, we will also ask all network members to complete a survey about the perceptions about the value of the network. The survey will include perceptions about the value, benefits and costs associated with being part of the network. This survey will be based on similar evaluations of implementation learning collaboratives [18, 19, 50]. Administration and data analysis procedures will follow those identified in the second stage.

## Discussion

This study protocol outlines the procedures for developing a network infrastructure/learning collaborative to facilitate the translation of research into practice. The overall goal of this infrastructure is to support Māori and Pacific community providers to help enhance health in their communities and thus help to address health inequities in Aotearoa [1, 3]. This infrastructure is an equity-oriented strategy with a focus on building capacity to help address inequities over time. Our 21-month “proof-of-concept” study will not be able to change inequities in that time period given its relatively small scale, but it should set the foundation for continued equity-oriented work.

Further, the focus on inequities in research reflects public discourses of a deficit model for Māori and Pacific peoples underpinned by limitations, weakness, and dependency [51]. Such thinking often directs focus to the communities and individuals as the problems needing to be fixed. We view the central discourse as needing to change, instead acknowledging that Māori and Pacific communities and community providers often hold the insights, knowledge and answers to address the inequities faced [29]. We have adopted a strengths-based approach highlighting the strengths of Māori and Pacific community providers and their respective communities for addressing the inequities from a whānau ora (extended family health and well-being) perspective. At the same time, the inequitable distribution of resources also indicates a need to build partnerships with various elements in the health and research systems, and have access to resources within these systems [31, 52].

There are several key elements to our approach. First, as guided by the HPW framework [31], we emphasise systems thinking and integrated knowledge translation. Systems thinking recognises the multiple factors and levels that shape health issues and takes a holistic perspective to address the complexity of local contexts [53]. Integrated knowledge translation focuses on co-production with end users in the implementation process to enhance sustainability, community benefit, and effectiveness [54]. We reflect these in the fact that the network will actively engage key members within the revised health system, particularly with the localities approach. We intend to link with the localities approach and other key organisations to support providers and reflect the larger changes and emphasis within the health system.

Second, further supporting the HPW framework, the construction of the network and administration of the implementation research procedures will be done in a strong community-engagement approach [55]. Our engagement process is reflective of Māori and Pacific worldviews and methodological approaches, and models of health and wellbeing, in that we centre provider experiences and knowledge and use a collaborative, participatory process for the implementation research and trials. The approach is this study reflects the Waitangi Tribunal 2575 principles of (a) the guarantee of tino rangatiratanga (self-determination); (b) the principle of partnership; (c) the principle of active protection; (d) the principle of equity; and (e) the principle of options. While these principles are treaty specific and geared toward Māori communities, our engagement with Pacific providers revealed that these principles also reflect their interests and those of their communities.

The study and infrastructure do have some limitations. Our population and geographical reach are modest as this is more of a “proof-of-concept” project than a direct reach and impact project per our specific aims and funding constraints. If the implementation network is successful on this scale, we can then look to expand and sustain it. We anticipate having 20-30 providers in the network with only 4–8 actually implementing trials so while we have the sufficient sample size to demonstrate effectiveness, we will not have the scope to have a large-scale impact. This is why we frame the study and an equity-oriented strategy. We do have sufficient evaluation efforts on implementation processes and outcomes, as well as evaluation research on the perceptions of members of the network.

In conclusion, this protocol outlines the development of a network that can support health equity efforts and the translations of research into practice in Aotearoa New Zealand. The participatory and culturally-centred approaches provide an opportunity to engage Māori and Pacific community providers in a collaborative manner. Including researchers and key members in the health system provides a robust infrastructure that has the potential for helping to the creative positive impact of research efforts rather than relying on individual research teams to development implementation plans and efforts in a piecemeal manner. A developed infrastructure may help a small country such as Aotearoa New Zealand create an efficient and effect manner to support its publicly funded health system.

## Supplementary Information

**Additional file 1.** Key Network Structures: Healthier Lives Implementation Research Network.

## Data Availability

The datasets used and/or analysed during the current study are available from the corresponding author on reasonable request.

## Abbreviations

CFIR: Consolidated Framework for Implementation Research
CTSA: Clinical and Translational Science Award
DHB: District Health Board
HPW: He Pikinga Waiora
LC: Learning collaborative
MHA: Māori Health Authority

## References

[1] Ministry of Health (2019). Wai 2575 Māori health trends report.

[2] Ministry of Health (2020). Annual update of key results 2019/20: New Zealand health survey.

[3] Walsh M, Grey C (2019). The contribution of avoidable mortality to the life expectancy gap in Māori and Pacific populations in New Zealand—a decomposition analysis. NZ Med J.

[4] Harris R, Cormack D, Tobias M, Yeh L-C, Talamaivao N, Minster J, Timutimu R (2012). The pervasive effects of racism: experiences of racial discrimination in New Zealand over time and associations with multiple health domains. Soc Sci Med.

[5] Reid P, Cormack D, Paine S (2019). Colonial histories, racism and health: the experience of Maori and Indigneous peoples. Public Health.

[6] Curtis K, Fry M, Shaban R, Considine J (2017). Translating research finding to clinical nursing practice. J Clin Nurs.

[7] Zurynski Y, Smith C, Knaggs G, Meulenbroeks I, Braithwaite J (2021). Funding research translation: how we got here and what do do next. Aust NZ J Public Health.

[8] Ammerman A, Smith TW, Calancie L (2014). Practice-based evidence in public health: improving reach, relevance, and results. Annu Rev Public Health.

[9] Morris Z, Wooding S, Grant J (2011). The answer is 17 years, what is the question? Understanding tmie lags in translation research. J R Soc Med.

[10] Chalmers I, Gaszious P (2009). Avoidable waste in the production and reporting of research evidence. Lancet.

[11] Smith J (2018). Towards a Kaupapa Māori Ako knowledge transfer system: Kāinga Tahi, Kāinga Rua.

[12] Glasgow R, Lichtenstein E, Marcus A (2003). Why don’t we see more translation of health promotion research to practice? Rethinking the efficacy-to-effectiveness transition. Am J Public Health.

[13] Greenlund KJ, Giles WH (2012). The Prevention Research Centers program: translating research into public health practice and impact. Am J Prev Med.

[14] Kaholokula J, Wilson R, Townsend C, Zhang G, Chen J, Yoshimura S, Dillard A, Yokota J, Palakiko D, Gamiao S (2014). Translating the diabetes prevention program in native Hawaiian and Pacific Islander communities: the PILI ‘Ohana project. Transl Behav Med.

[15] Baldwin L-M, Keppel G, Davis A, Guirguis-Blake J, Force R, Berg A (2012). Developing a practice-based research network by integrating quality improvement: challenges and ingredients for success. Clin Trans Sci.

[16] Gagliotti A, Werner J, Rust G, Fagnan L, Neale A (2016). Practice-based research networks (PBRNs): bridging the gaps between communities, funders, and policymakers. J Am Board Fam Med.

[17] Boulware LE, Corbie G, Aguilar-Gaxiola S, Wilkins CH, Ruiz R, Vitale A, Egede LE (2022). Combating structural inequities - diversity, equity, and inclusion in clinical and translational research. N Engl J Med.

[18] Evans L, Carter J, Costa M, Isenberg D, Procopio L (2021). Young S Strengths and challenges of implementing a learning collaborative in the Ryan White HIV/AIDS Program. Health Promot Pract.

[19] Nadeem E, Weiss D, Olin S, Hoagwood K, Horwitz S (2016). Using a theory-guided learning collaborative model to improve implementation of EBPs in a state children’s mental health system: a pilot study. Adm Policy Ment Health.

[20] Nix M, McNamara P, Genevro J, Vargas N, Mistry K, Fournier A, Shofer M, Lomotan E, Miller T, Ricciardi R (2018). Learning collaboratives: insights and a new taxonomy from AHRQ's two decades of experience. Health Aff.

[21] Green L, White L, Barry H, Nearse D, Hudson B (2005). Infrastructure requirements for practice-based research network. Ann Fam Med.

[22] Pronovost PJ, Berenholtz SM, Needham DM (2008). Translating evidence into practice: a model for large scale knowledge translation. BMJ.

[23] Damschroder LJ, Aron DC, Keith RE, Kirsh SR, Alexander JA, Lowery JC (2009). Fostering implementation of health services research findings into practice: a consolidated framework for advancing implementation science. Implement Sci.

[24] Kirk MA, Kelley C, Yankey N, Birken SA, Abadie B, Damschroder L (2016). A systematic review of the use of the Consolidated Framework for Implementation Research. Implement Sci.

[25] Harding T, Oetzel JG, Simpson ML, Nock S (2022). Identifying the facilitators and barriers in disseminating and adopting a health intervention developed by a community-academic partnership. Health Educ Behav.

[26] Simpson ML, Ruru S, Oetzel JG, et al. Adaptation and implementation processes of a culture-centered communitybased peer-education programme for older Māori. Implement Sci Commun. 2022; online. 10.1186/s43058-022-00374-3. 10.1186/s43058-022-00374-3 PMC9694883 36424640

[27] Oetzel JG, Hokowhitu B, Simpson ML (2019). Kaumtua Mana Motuhake: a study protocol for a peer education intervention to help Māori elders work through later-stage life transitions. BMC Geriatr.

[28] Simpson ML, Greensill H-M, Nock S (2020). Kaumātua mana motuhake in action: developing a culture-centred peer support programme for managing transitions in later life. Aging Soc.

[29] Hokowhitu B, Oetzel JG, Simpson ML (2020). Kaumātua Mana Motuhake Pōi: a study protocol for enhancing wellbeing, social connectedness and cultural identity for Māori elders. BMC Geriatr.

[30] Pronovost PJ, Berenholtz SM, Needham DM (2008). Translating evidence into practice: a model for large scale knowledge translation. BMJ.

[31] Oetzel J, Scott N, Hudson M (2017). Implementation framework for chronic disease intervention effectiveness in Māori and other indigenous communities. Glob Health.

[32] Simpson C, Oetzel JG (2019). Investigating the feasibility of establishing a practice-based research network in New Zealand.

[33] Health New Zealand. Future Of Health. https://www.futureofhealth.govt.nz/. Accessed 30 June 2022.

[34] Cresswell J (2009). Research design: qualitative, quantitative, and mixed methods approaches.

[35] Hintze J (2001). NCSS and PASS.

[36] Harding T, Oetzel J (2021). Implementation effectiveness of health interventions with Māori communities: a cross-sectional survey of health professional perspectives. Aust NZ J Public Health.

[37] Chaudoir S, Dugan A, Barr C (2013). Measuring factors affecting implementation of health innovations: a systematic review of structural, organizational, provider, patient, and innovation level measures. Implement Sci.

[38] Rarere M, Oetzel J, Masters-Awatere B (2019). Critical reflection for researcher-community partnership effectiveness: the He Pikinga Waiora process evaluation tool. Aust J Prim Health.

[39] Castaneda SF, Holscher J, Mumman MK (2012). Dimensions of community and organizational readiness for change. Prog Community Health Partnersh.

[40] Braun V, Clarke V (2006). Using thematic analysis in psychology. Qual Res Psychol.

[41] Oetzel J, Simpson M, Berryman K, Iti T, Reddy R (2015). Managing communication tensions and challenges during the end-of-life journey: perspectives of Māori kaumātua and their whānau. Health Commun.

[42] Oetzel J, Simpson M, Berryman K, Reddy R (2015). Differences in ideal communication behaviours during end-of-life care for Māori carers/patients and palliative care workers. Palliat Med.

[43] IBM Corp (2022). SPSS Statistics for Windows, version 30.

[44] Arbuckle J (2022). SPSS AMOS (version 30).

[45] RKCTRP Team (2012). Māori health literacy and communication in palliative care: Kaumātua-led models.

[46] Firestone R, Gaeamani G, Okiakama E (2021). Pasifika prediabetes youth empowerment programme: evaluating a co-designed community-based intervention from a participants’ perspective. Kōtuitui: NZ J Soc Sci Online.

[47] Verbiest M, Corrigan C, Dalhousie S (2019). Using codesign to develop a culturally tailored, behavior change mHealth intervention for indigenous and other priority communities: a case study in New Zealand. Transl Behav Med.

[48] Bernal G, Jiménez-Chafey MI, Domenech Rodríguez MM (2009). Cultural adaptation of treatments: a resource for considering culture in evidence-based practice. Prof Psychol Res Pract.

[49] Domenech Rodríguez MM, Baumann AA, Schwartz AL (2011). Cultural adaptation of an evidence based intervention: from theory to practice in a Latino/a community context. AM J Community Psychol.

[50] Haine-Schlagel R, Brookman-Frazee L, Janis B, Gordon J (2013). Evaluating a learning collaborative to implement evidence-informed engagement strategies in community-based services for young children. Child Youth Care Forum.

[51] Blakely T, Shilpi A, Bridget R, Martin T, Martin B (2004). Decades of disparity: widening ethnic mortality gaps from 1980 to 1999. NZ Med J.

[52] Dutta MJ (2007). Communicating about culture and health: theorizing culture-centered and cultural sensitivity approaches. Commun Theory.

[53] Frerichs L, Lich KH, Dave G, Corbie-Smith G (2016). Integrating systems science and community-based participatory research to achieve health equity. Am J Public Health.

[54] Grimshaw JM, Eccles MP, Lavis JN, Hill SJ, Squires JE (2012). Knowledge translation of research findings. Implement Sci.

[55] Yuen T, Park AN, Seifer SD, Payne-Sturges D (2015). A systematic review of community engagement in the US environmental protection agency’s extramural research solicitations: implications for research funders. Am J Public Health.

