# Supplementary material for: Healthier Lives Implementation Research Network for Māori and Pacific community health providers in Aotearoa New Zealand: a study protocol with an observational mixed methods design
Source: Implement Sci Commun. 2022 Nov 22;3:122. doi: 10.1186/s43058-022-00373-4 (PMC9686062; doi:10.1186/s43058-022-00373-4)
Supplement: Supplementary file 1 — Additional file 1. Key Network Structures: Healthier Lives Implementation Research Network. [file 43058_2022_373_MOESM1_ESM.docx]

## Key Network Structures: Healthier Lives Implementation Research Network

## Focus and Size

The overall goal of the project is to develop a network of community providers, researchers and health systems representatives that can help facilitate the implementation of novel programmes and products that help to meet the health needs of Māori and Pacific communities. More particular, our initial focus is to address hauora and community wellbeing through projects that emphasise prevention/early detection of various health conditions. The network will be community-provider focused with other members supporting the efforts of providers; providers will need to have strong community engagement approaches with their own communities. The network focus is to be further developed and refined through a co-design process with input from a variety of partners.

There are different levels of involvement of the network including just being informed of outputs, active trialling of outputs including an adaptation process to fit their communities and identifying additional research needs or needs for additional programmes. The network is a learning collaborative where all members agree to work in a participatory or collaborative manner to meet the needs of community providers to address health outcomes in their communities.

Initially, we are considering a single network with separate branches for Māori and Pacific communities to reflect their uniqueness. We think there are some common interests and philosophy that allow for a shared infrastructure and yet and can provide their own space for respective needs and interests. The size of the two branches is to be determined through the final co-design process, but initially we are thinking about having 10-15 Māori and Pacific community providers participating at an informational level and 2-4 doing active trialling (depending on the complexity of the implementation). We will invite providers from across the nation although active implementation will likely be limited to one or two regions to ensure sufficient resources for impact.

## Framework and Principles

The He Pikinga Waiora (Enhancing Wellbeing) Implementation Framework (HPW) provides the general framework for this project (See Figure 1).^6^ It outlines key principles for implementing health promotion programmes with Indigenous communities. HPW centres Indigenous perspectives and knowledge (such as Kaupapa Māori and Kaupapa Pasifika) and integrates four key elements: culture-centred approach, community engagement, systems thinking and integrated knowledge translation.

*Figure 1. He Pikinga Waiora Framework*

Each of these elements emphasise certain principles (italicised below) and are consistent with Te Tiriti o Waitangi and Pacific practices and philosophy:

- - 1. Indigenous mātauranga: *localised knowledge* and ways of knowing and doing (e.g., mana motuhake)
    2. Community engagement focuses on principles of *partnership and co-design* and *mutual respect*
    3. Culture-centredness identifies the need to have *localised knowledge and context* reflected in implementation and agency and *self-determination* in what works best for the community.
    4. System thinking: touchpoints with the system to provide support for implementation and *health equity*
    5. Integrated knowledge translation: *bidirectional learning and dialogue* and *sustainability*

Key Principles:

- *Localised knowledge and context*
- *Partnership and co-design*
- *Mutual respect*
- *Options and Self-determination*
- *Health equity*
- *Bi-directional learning and dialogue*
- *Sustainability*

## Network Structure

The structure of the network will include a leadership and administration team consisting of at least six members: three co-directors, two community researchers, and one communication/administrative support. One co-director (Oetzel) will be the central administrative lead responsible for overseeing communication, gathering information about potential programmes and liaising with touchpoints in the health system (including seeking funding). The other co-directors (Penetito-Hemara & Sika-Paotonu/Henry) will be responsible for the development and leadership of the Māori and Pacific branches of the network respectively. These two branches will be supported by a .75 FTE community researcher each. The community researcher roles may be split into several part-time positions depending on needs and availability of community researchers. The community researcher will help to construct the network and then work more closely to support the community providers during implementation of programmes. The communication/support person will be responsible for developing various methods for sharing information and helping to construct the learning collaborative culture of the network. These methods include Research briefs (2-page summaries of projects); b) Newsletter for sharing new programmes; c) Workshops (virtual and in-person) about programmes of interest, and d) E-mail and social media. These methods will be reviewed by network members and adaptations made to best meet preferences and needs.

Overall, the leadership team will support the development and functioning of the network in the following ways:

1. Recruiting network members and building trusting relationships with all members
2. Facilitating the co-design process in creating the network.
3. Gathering and sharing information about potential programmes for implementation
4. Conducting research to help identify lessons learnt about successful implementation
5. Providing support to community providers during the implementation process
6. Helping to develop research capacity to support implementation

The research focus will be co-constructed with network members and particularly the community providers. We will provide a starting framework and focus. We will explore the implementation process and outcomes to help identify what works and what does not during this process. We also hope to identify potential research questions and gaps and then put those questions out to the researchers in the network. (i.e., providers identify a need and no programmes exist).

The leadership team will be supported by an advisory group/steering group composed of approximately 6-8 members from various positions in the health system who can provide advice and also review evidence of programmes and recommend whether programmes should be trialled.

We also need to identify the appropriate touchpoints in the health system. We want to include key members in the health system as part of the network and also leverage existing efforts and projects to meet the needs of network members. These touchpoints will emerge during the co-design stage of the network.

Network members will include community providers, researchers, advisory board members, and other members in the health system. The next section outlines the benefits and expectations of these members.

## Benefits and Expectations

In response to the focus of value added and clear roles for participants, we developed a list of benefits and expectations for network members.

### Community Providers

*Benefits of Participating*

1. Learn about innovative programmes to improve health outcomes and health equity
2. Help identify key health needs that require the development of new programmes (i.e., help to set the research agenda and influence what researchers examine)
3. Opportunity to try out innovative programmes that you adapt to fit your local community needs while being supported from community and academic researchers (with limits depending on size of network and complexity of implementation)
4. Build research capacity around implementing new programmes
5. Learn from other providers about their experiences
6. Be part of a collective effort to improve health outcomes and health equity
7. Opportunity to advocate for funding of successful programmes and potentially receiving financial resources to support sustainable efforts

*Expectations*

1. Participate in the co-design of the network focus, needs, communication preferences and key principles to help establish the network (once per month to start)
2. Participate in quarterly meetings of the network to review needs and opportunities (one member per organisation)
3. Participate in webinars and in-person meetings around specific topics as identified by the network (per interest and availability)
4. Receive information about and review opportunities for novel health programmes
5. (For some, but not necessarily all member) Willingness to try out a new programme with financial and human resource support.
6. Identify local networks (e.g., schools, churches, Iwi/Hapū, community leaders, other partners) that can help support the implementation efforts and address hauora and community wellbeing

### Researchers

*Benefits of Participating*

1. Opportunity to share your innovative programmes and products with community providers
2. Increase the impact of your research by sharing your work
3. Share potential research opportunities and build partnerships
4. Opportunity to enhance engagement with community providers and key people in the health system
5. Be part of a collective effort to improve health outcomes and health equity

*Expectations*

1. Develop research briefs to identify the programme/product and evidence base supporting it
2. Share guidelines and experience with interested network members through a webinar, in-person meeting, or video summary
3. Work with interested network members to implement the programme (with support of the administrative team; i.e., the researcher will provide overall direction and the network team will provide support)
4. Work in a participatory manner with network members
5. Help to address research needs of the network through a collaborative process (including co-construction of research agenda and identifying funding opportunities)
6. (For some researchers, but not necessarily all) Participate in (quarterly or semi-annual) meetings of the network to review needs and opportunities
7. Participate in webinars and in-person meetings around specific topics as identified by the network (per interest and availability)

Other Kaupapa Partners in the Health System

*Benefits of Participating*

1. Help meet the needs of the health system, particularly in the context of the health reforms
2. Opportunity to enhance engagement with community providers and researchers
3. Be part of a collective effort to improve health outcomes and health equity
4. Support innovative programmes and translating research into practice and potentially policy

*Expectations*

1. Share funding opportunities, health system reforms and policy changes that may influence the work of the network
2. Help network members navigate the system of funding and sustaining implementation efforts that demonstrate effectiveness
3. Help evaluate the evidence base of implementation efforts
4. Work in a participatory manner with network members
5. Participate in quarterly meetings of the network to review needs and opportunities (one member per organisation)
6. Participate in webinars and in person meetings around specific topics as identified by the network (per interest and availability)

### Advisory Board Members

*Benefits of Participating*

1. Be part of a collective effort to improve health outcomes and health equity
2. Help meet the needs of the health system, particularly in the context of the health reforms
3. Opportunity to enhance engagement with community providers and researchers
4. Support innovative programmes and translating research into practice and potentially policy

*Expectations*

1. Participate in 2-4 meetings annually with the leadership team to review and provide advice about key the workings of the network (i.e., help steward the work of the network)
2. Evaluate the evidence base of proposed programmes to determine if there is sufficient evidence to support implementation in the network
3. Share funding opportunities, health system reforms and policy changes that may influence the work of the network
4. Work in a participatory manner with network members
5. Disseminate and advocate for the work of network members when evidence warrants
